# Implications of SCFAs on the Parameters of the Lipid and Hepatic Profile in Pregnant Women

**DOI:** 10.3390/nu13061749

**Published:** 2021-05-21

**Authors:** Maciej Ziętek, Zbigniew Celewicz, Justyna Kikut, Małgorzata Szczuko

**Affiliations:** 1 Department of Perinatology, Obstetrics and Gynecology Pomeranian Medical University in Szczecin, 2 Siedlecka Str., 72-010 Police, Poland; sekr.perinat@spsk1.szn.pl; 2 Department of Human Nutrition and Metabolomics, Pomeranian Medical University in Szczecin, 24 Broniewskiego Str., 71-460 Szczecin, Poland; justyna.kikut@pum.edu.pl (J.K.); malgorzata.szczuko@pum.edu.pl (M.S.)

**Keywords:** SCFAs, pregnancy, propionic acid, butyric acid, caproic acid, heptanoic acid, valeric acid

## Abstract

Short-chain fatty acids (SCFAs) are the product of the anaerobic intestinal bacterial fermentation of dietary fiber and resistant starch. An abnormal intestinal microbiota may cause a reduction in the production of SCFAs, which stimulate the development of intestinal epithelial cells, nourish enterocytes, influence their maturation and proper differentiation, reduce the pH, and are an additional source of energy for the host. There have been reports of the special role of SCFAs in the regulation of glucose and lipid metabolism during pregnancy. Aim: The aim of the study was to analyze the correlation of SCFAs with lipid and hepatic metabolism during pregnancy in relation to the body weight of pregnant women. Material and methods: This study was conducted in pregnant women divided into two groups: Obese (OW—overweight and obese women; *n* = 48) and lean (CG—control group; *n* = 48) individuals. The biochemical plasma parameters of lipid metabolism (TG, CH, LDL, HDL), inflammation (CRP), and liver function (ALT, AST, GGT) were determined in all of the subjects. SCFA analysis was performed in the stool samples to measure acetic acid (C 2:0), propionic acid (C 3:0), isobutyric acid (C 4:0 i), butyric acid (C 4:0 n), isovaleric acid (C 5:0 i) valeric acid (C 5:0 n), isocaproic acid (C 6:0 i), caproic acid (C 6:0 n), and heptanoic acid (C 7:0). Results: Statistically significant differences in the concentrations of C 3:0 and C 6:0 n were found between women in the OW group compared to the CG group. The other SCFAs tested did not differ significantly depending on BMI. The C 2:0, C 3:0, and C 4:0 n ratios showed differences in both OW and CG groups. In the OW group, no relationship was observed between the concentrations of the SCFAs tested and CRP, ALT, AST. A surprising positive relationship between C 5:0 n and all fractions of the tested lipids and branched C 5:0 with CHL, HDL, and LDL was demonstrated. In the OW group, HDL showed a positive correlation with C 3:0. However, lower GGT concentrations were accompanied by higher C 4:0 and C 5:0 values, and this tendency was statistically significant. Conclusions: The results of our research show that some SCFAs are associated with hepatic lipid metabolism and CRP concentrations, which may vary with gestational weight. Obesity in pregnancy reduces the amount of SCFAs in the stool, and a decrease in the level of butyrate reduces liver function.

## 1. Introduction

Lipid metabolism includes the biosynthesis and degradation of lipids such as fatty acids, triglycerides, and cholesterol. Maternal lipids are a source of fractions necessary for the growth and development of the fetus, which has a limited capacity for de novo lipogenesis and fatty acid oxidation. Although lipid metabolism during physiological pregnancy has been well documented, the role of SCFAs in this period is still poorly understood. Placental free fatty acid (FFA) uptake from the maternal bloodstream delivers fatty acids to both placental and fetal metabolism via fatty acid binding proteins (FABP) [1]. Among the types of FABP (i.e., FABP1, FABP3, FABP4, FABP5) present in trophoblasts [2], obese women exhibit a marked increase in FABP4 expression, which leads to changes in lipid content. The significant changes in methylation observed during early pregnancy and the relative hypomethylation of the placenta indicate that it may be susceptible to diet intervention. Thus, fetal epigenetic modifications and fetal susceptibility to obesity may result from both the metabolic environment and the diet during pregnancy [1]. Recently, the relationship between the populations of intestinal microorganisms, the production of SCFAs, FFA levels, and mediators activating the immune system’s defense mechanisms, such as interleukin-6 (IL-6) and monocyte chemoattractant protein-1 (MCP-1) interferon-γ (IFNγ), has been emphasized [3].

### 1.1. SCFAs in Pregnancy

SCFAs are carboxylic acids with aliphatic tails of one to six carbons. As products of bacterial anaerobic digestion of dietary fiber in the large intestine, SCFAs not only play an energetic role [4], but also act as signaling molecules via specific G-protein-coupled receptors (GPCRs), transmitting messages between the microbiota and the immune system [5]. SCFAs serve, inter alia, the de novo synthesis of lipids and glucose, which are the host’s main source of energy [6].

In turn, during the fermentation of branched amino acids (valine, leucine, and isoleucine) by the intestinal microflora, branched-chain fatty acids (BCFA) are produced, which are saturated fatty acids with one or more methyl branches in the carbon chain. In the human intestine, branched chain amino acid fermentation is mainly carried out by the genera *Bacteroides* and *Clostridium* [7], and BCFA levels show increasing concentrations from proximal to distal colon and feces. BCFAs, which mainly include isovaleric and isobutyric acids, are produced in much lower amounts than SCFAs, and their levels in the feces of various human groups, gut-producing populations, and health effects are not yet sufficiently understood [8]. It has been shown that the molar proportions of BCFA in the feces show a strong positive correlation with increasing age [8]. The amount of SCFAs produced in the digestive tract varies and depends on the diet, type, and host microbiome capacity, and the time of residence in the digestive tract [8,9].

The most common metabolites in studies of the microbiome in pregnancy are butyric, acetic, and propionic acids, which are present in a certain molar ratio, which may change depending on the diet, age, and treatment of diseases, among other factors. The concentration of SCFA varies depending on their location: In the distal part of the colon, the concentration is lower (20–70 mM) than in the proximal part (70–140 mM), which has higher availability of carbohydrates and water [9]. It is suspected that acetic and propionic acids, in combination with L-lactate, play an important role in the regulation of lipid and cholesterol metabolism [10], and acetic acid is also a primary substrate in the synthesis of cholesterol [11,12]. SCFAs are also responsible for maintaining the balance of anti- and pro-inflammatory responses, acting as a kind of messenger between the intestinal commensal consortium and the immune system. In an animal model, it has been shown that the concentrations of acetic and propionic acids increase during pregnancy [13].

### 1.2. Effect of SCFAs on the Lipid Profile

SCFAs directly activate 5′AMP-activated protein kinase (AMPK), the activation of which in skeletal muscles inhibits the synthesis of glycogen and proteins and supports glucose transport and fatty acid oxidation [4]. While lipid oxidation is activated by SCFA, de novo lipid synthesis and lipolysis are inhibited [14]. SCFA does not appear to influence lipolysis of fatty acids in the liver, while lipolysis in adipose tissue is strongly reduced by SCFA [15]. In isolated adipocytes, acetic and propionic acids inhibit lipolysis by activating the free fatty acid receptor 2 (Ffar2). Thus, the prevention of diet-induced obesity by SCFA can be attributed to increased fatty acid oxidation in many tissues and a reduction in fat stored in white adipose tissue. Lipid metabolism and inflammation play a critical role during pregnancy [8]. The fat deposition process observed during pregnancy is associated with an increase in the concentration of phospholipids, non-esterified fatty acids, and triglycerides (TG) in the maternal circulation. The mechanism responsible for this process is the reduction of lipoprotein lipase (LPL) activity in adipose tissue and an increase in insulin resistance [12]. Under physiological conditions, one of the mechanisms by which the body is protected from excessive lipid intake and diet-induced obesity is an elevated fasting fat factor (FIAF) [16]. FIAF inhibits the action of LPL, the enzyme responsible for energy storage in the form of fat [17]. Accelerated lipid metabolism and the formation of ketone bodies are a response characteristic of diabetes, long fasting periods, and pregnancy. In the first half of pregnancy, the storage of adipose tissue increases and then gradually decreases up to gestation at term. The concentration of all three lipoprotein fractions—very low density lipoproteins (VLDL), low density lipoproteins (LDL), and high density lipoproteins (HDL)—increases. Apart from pregnancy, with the increase in insulin production, a more intense action of lipoprotein lipase in fat cells is observed, leading to the hydrolysis of TG to free fatty acids and glycerol [18].

Although long-chain polyunsaturated fatty acids (LC-PUFA) play a key metabolic role during pregnancy [19], SCFAs appear to be equally important for their role in inflammation, as well as for obesity associated with pro-inflammatory adipose tissue status. In an animal model, obese females showed a reduced relative abundance of several types of bacteria belonging to the linear buttery-producing families, as evidenced by a reduced concentration of C 4:0n in the mother’s cecum, SCFA receptor transcripts (Ffar2, Ffar3, Hcar2—Hydroxycarboxylic Acid Receptor 2), and Muc2 mucosal proteins [19].

It has been shown that maternal diet-induced obesity is associated with the reduction of genes encoding the tightly linked proteins claudin-1 and zonula occludens-1 (ZO-1) in the maternal intestine, which is the most important factor in the regulation of intestinal permeability. This observation suggests that for diet-dependent obesity, SCFAs and their receptors lose their protective effect on the maternal gut barrier function [20,21]. A high-fat diet (HFD) has been associated with a reduction in the relative abundance of SCFA-producing bacteria during pregnancy [22]. On the other hand, an obesity-inducing diet in pregnant women increased intestinal pro-inflammatory activity through the maternal NFκB pathway, colon CD3 + T cell count, and placental inflammation, thus impairing maternal bacterial metabolite signaling, which ultimately generates significant structural changes in the placental blood vessels [19].

The microbiota is also an important factor affecting circulating lipid levels including TG and HDL. Vojinovic et al. [23] demonstrated the association of 32 families and microbial genera with VLDL and HDL subfractions, serum lipid concentrations, glycolysis-related metabolites, ketone bodies, amino acids, and acute phase reaction markers.Obesity and pregnancy may affect the quantity of SCFAs. The possible mechanism that explains this effect is based on acetate use as a substrate in cholesterol synthesis, which may cause a reduction in the number of butyric acid synthesizing bacteria, decreasing lipolysis, and increasing fatty acid oxidation and fat storage in white adipose tissue. A decrease in butyrate production leads to maternal intestinal barrier dysfunction through the NFκB pathway activation.

### 1.3. Effect of BCFA on the Lipid Profile

Fecal BCFA levels have been found to be higher in people with hypercholesterolemia compared to people with normocholesterolemia, with fecal isobutyric acid levels associated with a poorer serum lipid profile [24]. This indicates that there may be a relationship between BCFA and lipid metabolism. In vitro studies have shown that BCFAs inhibit both cAMP-mediated lipolysis and insulin-stimulated lipogenesis in adipocytes, while isobutyric acid has the effect of promoting cellular insulin-induced glucose uptake [25].

### 1.4. Effect of SCFAs on the Hepatic Profile

The effect of SCFA supplementation on the dynamics of lipoprotein synthesis in humans has not been studied. In an animal model, it has been shown that a mixture of different SCFAs (sodium salts of acetic, propionic, and butyric acids) administered to rats in the diet reduced the rate of cholesterol synthesis, which probably contributed to reduced plasma cholesterol levels [26]. Dysbiotic microbiome disorders are associated with the occurrence of liver diseases such as non-alcoholic steatosis (NAFLD) [27]. It has been shown in a rat model that butyric acid maintains the intestinal barrier by reversing abnormal ZO-1 expression and reducing endotoxin translocation. Its hepatoprotective effect is particularly evident in total hepatic ischemia reperfusion injury [28]. Juanola et al. [29] confirmed the negative correlation of butyric acid with inflammatory markers and serum endotoxins, suggesting that lower blood SCFA levels in patients with cirrhosis is associated with more advanced liver disease. Thus, a lower proportion of this SCFA is associated with liver disease progression. In turn, the action of propionic acid may be associated with liver damage, which has been demonstrated in an animal model involving rats [30]. Despite the poorly expressed presence of SCFAs in the liver, their concentrations are further reduced in the hepatic circulation in advanced liver diseases (steatosis), which suggests their metabolic involvement in these disorders [29].

## 2. Material and Methods

### 2.1. Study Group

We analyzed prospectively collected data from 96 pregnant women aged 28–36 years who were consulted at the Department of Perinatology, Obstetrics and Gynecology Pomeranian Medical University in Szczecin, Poland. The diagnosis of pregnancy was based on obstetric interview and high-resolution ultrasound examination (ultrasound system Voluson E8, GE Healthcare, Austria GmbH & Co. OG; Vienna). Women past week 10 of gestation were enrolled in the study. The exclusion criteria were twin pregnancies, refusal to consent to examination, presence of an active infection or neoplastic disease, pregnancies complicated with metabolic diseases, and other high-risk pregnancies. Due to the possible influence of body weight and the related metabolic state on the examined lipid parameters and SCFAs, women were divided into two groups, depending on their BMI before pregnancy. To standardize the measurements of body weight and height (with an accuracy of 0.1 kg and 0.5 cm, respectively), all measurements were performed using a digital body weighing scale equipped with high precision sensors (Detecto PD200, New York; USA). To increase the accuracy of the measurements taken, the weight and height measurement equipment was calibrated after each use, measurements were taken by trained physicians, and measurements were repeated twice. A protocol was followed regarding the removal of clothing and/or jewelry, and participants emptied their bladder and/or bowels prior to measurement. The first group consisted of 48 overweight and obese (OW) women (BMI > 30), while the second one was a control group (CG) that consisted of 48 women with normal body weight, as shown in Table 1.

### 2.2. Determination of Biochemical Blood Parameters

Blood samples (5 mL) were collected from the antecubital vein of women who had fasted for 10 h since their last meal. Biochemical parameters of lipid metabolism (TG, CHL, LDL, HDL), inflammation (CRP), and liver function (ALT, AST, GGT) were measured by standard laboratory methods in an accredited diagnostic laboratory using Roche Diagnostic Cobas e411 and Cobas Integra 400 Plus analyzers.

### 2.3. SCFA Isolation

A mixture of a 0.5 g stool sample suspended in 5 mL of distilled water was intensively mixed for 5 min with the use of a shaker. With use of HCl solution, the pH of the suspension was adjusted to pH 2–3. The samples were then shaken for 10 min and centrifuged for 20 min at 5000 rpm. The supernatant was then filtered (Ø 400 µm filter) and transferred to a chromatography vial for gas chromatographic analysis.

### 2.4. SCFA Gas Chromatography

The following SCFAs were included in the analysis: acetic acid (C 2:0), propionic acid (C 3:0), isobutyric acid (C 4:0 i), butyric acid (C 4:0 n), isovaleric acid (C 5:0 i), valeric acid (C 5:0 n), isocaproic acid (C 6:0 i), caproic acid (C 6:0 n), and heptanoic acid (C 7:0). Chromatographic analyses were performed using an Agilent Technologies 1260 A GC system with a flame ionization detector (FID). A fused silica capillary column with a free fatty acid phase (DB-FFAP, 30 m × 0.53 mm × 0.5 µm) was used. The carrier gas was hydrogen with a flow rate of 14.4 mL/min. The heating process was initiated with a temperature of 100 °C for 0.5 min, which was then raised to 180 °C with an incremental rise of 8 °C/min. Following this, the temperature was increased to 200 °C over 1 min, and this temperature was maintained for 5 min. A volume of 1 μL sample extract was used and the run time of a single analysis was 17.5 min. Fatty acids were identified by comparing their retention times with commercially available standards [30].

### 2.5. Statistical Analysis

Statistical analysis was performed using Statistica 13 (Statsoft, Kraków, Poland). The standard parametric Shapiro–Wilk test was used to test the normality of the data. The distribution of most variables used in the analysis was normal. Non-parametric Mann–Whitney tests were used for comparisons between groups (obese women before pregnancy and the control group), and a *p*-value of <0.05 was considered statistically significant. The correlations of SCFAs with CRP concentrations, biochemical parameters of lipid and liver metabolism, and anthropometric measurements in women were also calculated.

## 3. Results

### 3.1. Fecal SCFA Percentage by Group Division

**Figure 1:**
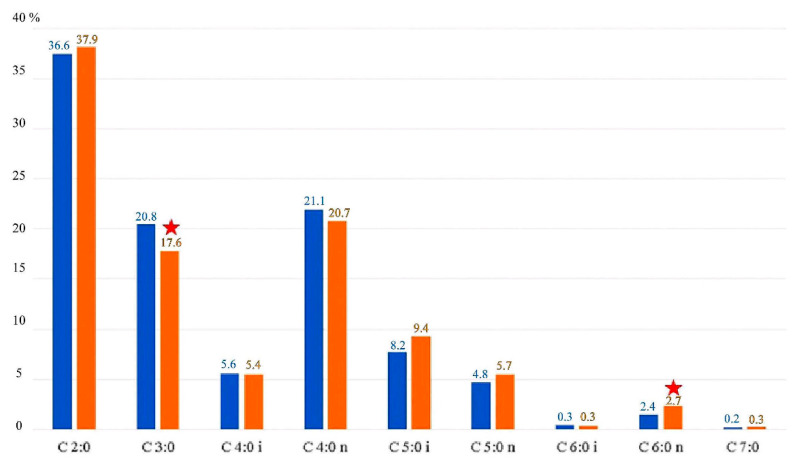
SCFAs in the experimental group (OW) and control group (CG) expressed as a percentage. OW—blue color; CG—orange color; ★ indicates statistically significant differences (*p* < 0.05). C 2:0—acetic acid; C 3:0—propionic acid; C 4:0 i—butyric (branched) acid; C 4:0 n—butyric (linear) acid; C 5:0 i—valeric (branched) acid; C 5:0 n—valeric (linear) acid; C 6:0 i—caproic (branched) acid; C 6:0 n—caproic (linear) acid; C 7:0—heptanoic acid.

The concentrations of individual SCFAs determined in the feces showed significant differences. The most common SCFA in all of the analyzed groups was acetic acid (37.23%), followed by butyric acid (21.1%) and propionic acid (17.5%). There were statistically significant differences in concentrations of propionic and caproic acids between women from the OW and the CG group. The other SCFAs tested did not differ significantly depending on BMI (Figure 1).

### 3.2. Correlations of SCFAs with Metabolomic Parameters in the Control Group (CG)

The level of plasma CRP was positively correlated with acetic, propionic and butyric (linear) acid levels. With regard to the serum liver enzyme levels, the ALT levels were positively correlated with isocaproic acid levels and were negatively associated with caproic acids levels. GGT levels were also negatively correlated with caproic acids levels. There were no significant differences in the AST levels in the CG (Table 2). The level of different lipid fractions (LDL, HDL, TG, CHL) was similar in both the OW and CG groups (Table 1). However, there was a positive correlation between acetatic, propionic, and butyric acids and CHL, HDL, and TG (Table 2). Acetic and butyric acid also exhibited a positive correlation with LDL, while the relationship between LDL and propionic acid may be described as a trend in the control group (Table 2).

### 3.3. Correlations of SCFA with Metabolic Parameters in the OW

In the OW group, no relationship was observed between the concentrations of the SCFAs tested and CRP, ALT, and AST (Table 3). However, there were unexpected positive correlations between linear valeric acid with all fractions of tested lipids, and between isovaleric acid and CHL, HDL, and LDL. In the group of obese and overweight women, HDL also showed a positive correlation with propionic acid. Lower GGT concentrations were accompanied by higher values of isobutyric, valeric, and isovaleric acids, and this trend was statistically significant (Table 3).

## 4. Discussion

Under normal physiological conditions, the proportions of the individual SCFAs most often remain constant with a molar ratio of 60:20:20 for acetic, propionic and butyric acids, respectively [12]. In our study, the molar ratios of acetic, propionic and butyric acid and other SCFAs showed differences both in the group of obese women (37:21:27:13:2, respectively) and those with normal body weight (38:18:26:15:3, respectively). We found that acetic acid is the dominant SCFA regardless of body weight, which is consistent with findings of other studies [4,31].

Increasing the amount of soluble fiber in the diet has been shown to contribute to the increase of SCFAs in the intestine [32,33], while a high-fat diet with a low dietary fiber content lowers levels of SCFAs [34]. The composition of the gut microbiota changes during pregnancy [35,36], and this may be of key importance for the composition of SCFAs. It is suggested that the pregnancy influences the abundance of specific bacterial phyla in gut of mice. During human pregnancy, the structure of the gut microbiota also changes significantly, with a reduction in species richness from trimester one to three [13]. A positive effect on the growth of SCFAs through their increased production can be obtained through changes to the diet [13]. Diet seems to be a key element in regulating the content of SCFAs during pregnancy [37]. In a study by De Fillipo et al. [34], it was shown that the predominantly dietary fiber diet consumed by African communities significantly increases the content of SCFAs in their stools [34]. A short-term change in diet changes the stool bacterial composition over a short time and reversibly, while a long-term change in diet is associated with irreversible changes [37]. The radical change from a high-fiber diet to a high-animal diet in just five days changes the number of bacteria in the gut, with a visible reduction in Firmicutes strains [38].

Studies have also shown that individual SCFAs play an important metabolic role; for example, acetic acid supplementation reduces weight gain and improves glucose tolerance in obese individuals and diabetic rats [39], butyric acid protects against obesity and increases thermogenesis in mice [40], and propionic and butyric acids improve glucose homeostasis in mice [41]. Priyadarshini et al. [42] conducted a study on a small cohort of pregnant obese and non-obese women and investigated the relationship between SCFAs levels during pregnancy with key metabolic parameters in mothers and newborns. They found that serum acetate levels were associated with maternal weight gain and maternal adiponectin levels. However, considering the small group of women included (n = 20), the above conclusions are debatable and need to be confirmed with a larger sample size.

Changes to the intestinal microbiota disturbs not only metabolism but also the composition of the host’s lipids by interacting with the diet [43]. Under normal physiological conditions, over the duration of a pregnancy there is a visible increase in lipid concentrations, including in TG and CHL [44]. Adank et al. [45] conducted a study on pregnant women and found a correlation between the concentrations of TG and remnant cholesterol in early pregnancy and increased fetal body weight and risk of having large for gestational age (LGA) infants. It has been shown in mice that circulating TG, HDL, and total CHL are increased by the gut microbiota under conditions of a high-fat diet [46]. One of the theories justifying the above dependence is the observation of the induction of liver production of monounsaturated fatty acids by the intestinal microbiota and the elongation of PUFA, and the acetic acid produced by the intestinal microflora is used as a precursor of the synthesis of hepatic fatty acids [47]. The introduction of a low-energy diet increases the quantity and richness of the assayed microorganisms which, through SCFAs, downregulate serum lipid levels [48]. Le Chantelier et al. [49] showed that TG was higher and HDL levels were lower in people with lower microbial counts compared to those with high microbial counts. As a result of a diet rich in fiber, the absorption of bile acids and cholesterol is inhibited, and CHL synthesis is suppressed, resulting in a reduction in serum CHL concentrations [26]. In our study, we observed a positive correlation between acetic, propionic, and butyric acid levels and CHL HDL, LDL, and TG levels in the control group of healthy women. In a study by Granado-Serrano et al. [24], the concentrations of acetic and propionic acid in the serum did not differ significantly between individuals with hypercholesterolemia (HC) and normocholesterolemia (NC), but they did find a tendency toward higher acetic acid concentrations and lower propionic acid concentrations in the HC and NC, respectively. This dependence, partially confirmed in our research, proves the relationship between SCFAs produced by specific bacteria in the intestine and lipid metabolism during pregnancy.

It has been shown that the composition of gut bacteria in humans with hypercholesterolemia is characterized by lower amounts of *Anaeroplasma* (Tenericutes) and *Haemophilus* (Proteobacteria), and higher amounts of *Odoribacter* (Bacteroidetes) and *Ruminococcus* (Firmicutes) compared to subjects with normal cholesterol levels [24]. Another study found a positive correlation between *Collinsella* species and insulin, TG, and VLDL levels. On the other hand, *Collinsella* tended to be negatively correlated with maternal HDL cholesterol. However, the authors did not find any relationship with LDL [50]. In our study, among the analyzed SCFAs, acetic, propionic, and butyric acid exhibited a relationship with the lipid profile in lean women, while valeric acid exhibited a relationship with obese women. These results may support the hypothesis that acetic acid circulating in the blood serum is associated with de novo lipogenesis and stimulation of hepatic cholesterogenesis, while propionic acid may inhibit them [51]. Administration of acetic acid has been shown to affect both total body lipolysis as well as intracellular lipolysis in adipocytes in in vitro and in vivo animal and human studies [52]. The antilipolytic effect of acetic acid results in a reduction in lipid transfer to insulin-sensitive peripheral tissues (e.g., skeletal muscle), which may improve insulin sensitivity and reduce hypothalamic inflammation. Two main mechanisms concerning the influence of the intestinal microflora on the lipid metabolism and fat reserves in pregnancy have been proposed: Increased efficiency of energy extraction from the diet, and unfavorable differentiation of the host microbiota, instead favoring metabolic inflammation [35]. In this study, no significant correlation between butyric acid and the lipid profile was shown in obese women. These findings are consistent with other studies where the stool butyric acid content did show no significant differences in HC and NC groups of patients [24].

A study by Goffredo et al. [53] showed a positive correlation between serum acetic, propionic, and butyric acids with body weight (BMI), fat content in subcutaneous tissue, and de novo hepatic lipogenesis. They also found that the Firmicutes to Bacteroidetes ratio index, and the content of Bacteroidetes and Actinobacteria were related to BMI and fat content in subcutaneous tissue. The gut microflora in young obese people is likely to be more capable of oxidizing dietary carbohydrates than the gut flora of lean people. People whose bodies oxidize sugars faster than lipids are therefore more likely to become obese than people who oxidize fats first.

In the next part of our study, an attempt was made to link changes in SCFAs concentrations with lipid metabolism and liver function. In studies by Shimizu et al. [54], dietary SCFAs were shown to inhibit liver weight and lipid synthesis. At the same time, supplementation with SCFAs improved the metabolic function of the liver through FFAR3, without affecting the intestinal environment. In the diagnosis of liver function, the enzymes AST, ALT, and GGT were considered. GGT is a marker used routinely in the diagnosis of liver and biliary diseases, and its activity increases in acute pancreatitis and primary pancreatic tumors [54]. GGT is a particularly sensitive diagnostic indicator in cholestasis, alcoholic liver disease, and liver cancer. Feng et al. [55] showed a relationship between the GGT to HDL cholesterol index with the occurrence of non-alcoholic fatty liver disease (NAFLD) and the metabolic syndrome. GGT activity is higher in overweight and obese women, which confirms its association with anthropometric parameters, insulin resistance, and metabolic syndrome in young women [56]. Significant differences in GGT concentrations in women with IR, regardless of BMI, may be of importance during pregnancy with physiological IR. In obese women, GGT is positively correlated with TC, LDL-C, TG, and the TC/HDL-C ratio [57]. The negative correlation of GGT with isobutyric and valeric acids observed in our study in obese women confirmed the relationship between obesity and SCFAs during pregnancy. A reduction of GGT in response to increasing concentrations of butyric acid proved its protective role in the liver. There are reports that butyric acid supplementation may also improve motor functions at the nervous system level, presenting a high potential to affect brain physiology [58].

The main limitation of this study was the small number of women included in the sample. A study conducted on a large group of women would allow for more objective conclusions to be reached. Undoubtedly, an analysis of the microbiome profile of each study participant in relation to SCFAs and the biomarkers studied would have been a beneficial enrichment of our study. Although no firm conclusions can be drawn based on the analysis of the literature and the studies conducted to date, the existence of associations between nutrient intake, the gut microbiome, and SCFA populations has recently been highlighted. However, in our study, no samples of the gut microbiota of the women studied were collected and therefore the hypothesis that individual bacteria have an influence on biomarkers of the lipid and liver profile through the production of SCFAs could not be directly considered. Furthermore, to date, the contribution of SCFAs of intestinal origin or from other production sources has not been well studied. Despite the extensive knowledge that has been accumulated on lipid and liver metabolism during pregnancy, there are still a considerable number of open questions that are currently being investigated in several research projects. Little is known about the implications of maternal SCFAs, lipids, and liver markers under healthy or pathological conditions and their impact on fetal development as well as short- and long-term health consequences.

## 5. Conclusions

A significant number of reports on the role of SCFAs involve an animal model, making it difficult to draw valid conclusions for the human system. Our results indicate that some SCFAs are associated with hepatic lipid metabolism and may vary with gestational weight. Statistically significant differences in propionic and caproic acids concentrations were found between obese and lean pregnant women. The correlation analysis performed in our study showed that some lipid fractions (CHL, HDL, LDL, TG) and CRP levels are significantly and positively correlated with acetic, propionic, and butyric acids in the group of lean pregnant women (CG). Lower GGT concentrations were accompanied by higher isobutyric and valeric acids levels, and this trend was statistically significant in the overweight and obese pregnant women (OW). Although the small study group in our work is a limitation for drawing definitive conclusions, the correlations shown despite small but statistically significant differences may provide directions for conducting further studies in a larger group of pregnant women. The low statistical power and the lack of size effect indicated by meta-analysis conducted in humans may have prevented the identification of additional factors associated with links between SCFAs and liver/lipid metabolism.

## Figures and Tables

**Table 1:** Demographic, anthropometric, and biochemical features of OW and CG subjects. The mean and standard deviation of each variable are shown.

Parameter	OW Mean ± SD	CG Mean ± SD	*p*-Value
Age (years)	30.76 ± 5.50	33.72 ± 4.96	NS
Height (m)	1.69 ± 0.06	1.66 ± 0.05	NS
Body weight before pregnancy (kg)	99.58 ± 14.30	65.32 ± 8.94	<10^−6^
Body weight during pregnancy (kg)	102.25 ± 14.77	71.28 ± 10.02	<10^−6^
BMI before pregnancy (kg/m^2^)	34.18 ± 7.50	22.06 ± 6.01	<10^−6^
BMI during pregnancy (kg/m^2^)	35.07 ± 7.64	24.82 ± 5.27	<10^−6^
Weight gain (kg)	2.61± 4.26	7.88 ± 10.49	<10^−6^
Week of gestation (week)	20.19± 6.10	23.13 ± 7.51	NS
CHL (mg/dL)	209.56 ± 40.83	224.0 ± 46.91	NS
HDL (mg/dL)	66.37 ± 13.78	77.77 ± 12.81	NS
LDL (mg/dL)	130.44 ± 38.72	140.43 ± 40.76	NS
TG (mg/dL)	178.42 ± 75.96	161.06 ± 58.42	NS
AST (IU/L)	16.65 ± 7.37	17.41 ± 7.92	NS
ALT (IU/L)	18.26 ± 12.34	18.88 ± 16.74	NS
GGT (IU/L)	13.26 ± 10.93	10.09 ± 10.44	NS

Abbreviations used: BMI—body mass index; LDL—low density lipoprotein; HDL—high density lipoprotein; CHL—total cholesterol; TG—triglycerides; AST—aspartate transaminase; ALT—alanine aminotransferase; GGT—gamma-glutamyl transferase; NS—not significant.

**Table 2:** Correlation coefficients indicating the extent to which SCFAs in the stool and the examined plasma parameters in the CG are interdependent.

CG	CRP (mg/dL)	ALT (IU/L)	CHL (mg/dL)	HDL (mg/dL)	LDL (mg/dL)	TG (mg/dL)	GGT (IU/L)
C 2:0	0.997	NS	0.964	0.951	0.950	0.925	NS
C 3:0	0.903	NS	0.914	0.949	NS	0.933	NS
C 4:0 i	NS	NS	NS	NS	NS	NS	NS
C 4:0 n	0.951	NS	0.999	0.988	0.998	0.985	NS
C 5:0 i	NS	NS	NS	NS	NS	NS	NS
C 5:0 n	NS	NS	NS	NS	NS	NS	NS
C 6:0 i	NS	0.958	NS	NS	NS	NS	NS
C 6:0 n	NS	−0.913	NS	NS	NS	NS	−0.955
C 7:0	NS	NS	NS	NS	NS	NS	NS

C 2:0—acetic acid; C 3:0—propionic acid; C 4:0 i—butyric (branched) acid; C 4:0 n—butyric (linear) acid; C 5:0 i—valeric (branched) acid; C 5:0 n—valeric (linear) acid; C 6:0 i—caproic (branched) acid; C 6:0 n—caproic (linear) acid; C 7:0—heptanoic acid; CRP—C-reactive protein; ALT—alanine aminotransferase; CHL—total cholesterol; HDL—high density lipoprotein; LDL—low density lipoprotein; TG—triglycerides; GGT—gamma-glutamyl transferase; NS—not significant.

**Table 3:** Correlation coefficients indicating the extent to which SCFAs in the stool and the examined plasma parameters in the OW are interdependent.

OW	CHL (mg/dL)	HDL (mg/dL)	LDL (mg/dL)	TG (mg/dL)	GGT (IU/L)
C 2:0	NS	NS	NS	NS	NS
C 3:0	NS	0.725	NS	NS	NS
C 4:0 i	NS	NS	NS	NS	−0.622
C 4:0 n	NS	NS	NS	NS	NS
C 5:0 i	0.654	0.603	0.656	NS	−0.644
C 5:0 n	0.733	0.754	0.663	0.667	−0.678
C 6:0 i	NS	NS	NS	NS	NS
C 6:0 n	NS	NS	NS	NS	NS
C 7:0	NS	NS	NS	NS	NS

C 2:0—acetic acid; C 3:0—propionic acid; C 4:0 i—butyric (branched) acid; C 4:0 n—butyric (linear) acid; C 5:0 i—valeric (branched) acid; C 5:0 n—valeric (linear) acid; C 6:0 i—caproic (branched) acid; C 6:0 n—caproic (linear) acid; C 7:0—heptanoic acid; CHL—total cholesterol; HDL—high density lipoprotein; LDL—low density lipoprotein; TG—triglycerides; GGT—gamma-glutamyl transferase; NS—not significant.

## Data Availability

Data available on request.
